# Macrogenomics reveal the effects of inter-cropping perilla on kiwifruit: impact on inter-root soil microbiota and gene expression of carbon, nitrogen, and phosphorus cycles in kiwifruit

**DOI:** 10.3389/fmicb.2024.1349305

**Published:** 2024-06-03

**Authors:** Ning Gao, He Wen, Zhiwei Shang, Yifei Zou, Wei Zhao, Yun He, Sen Yang, Heng Zhang, Jiahao Qin, Sixi Zhu, Wenhua Wang

**Affiliations:** ^1^Guizhou Rapeseed Institute, Guizhou Academy of Agricultural Sciences, Guiyang, China; ^2^Guizhou Minzu University, Guizhou, China

**Keywords:** kiwifruit, perilla, carbon, nitrogen and phosphorus cycling genes, inter-root microorganisms, macrogenome, inter-cropping

## Abstract

Intercropping systems can improve soil fertility and health, however, soil microbial communities and functional genes related to carbon, nitrogen and phosphorus cycling under the intercropping system of mesquite and perilla have not been studied. Therefore, in the present study, different planting densities and varieties of *Perilla frutescens* (L.) Britt and kiwifruit were used for intercropping, and changes in soil microbial communities and carbon, nitrogen, and phosphorus cycling genes in kiwifruit inter-roots under inter-cropping conditions were investigated by macro-genome sequencing technology. The results showed that intercropping with Perill caused a decrease in most soil nutrients, soil enzyme activities, and had a significant impact on the microbial (bacteria and fungi) diversity. Inter-cropping increased the relative abundance of the dominant bacterial phylum “Proteobacteria” and “Actinobacteria” by 47 and 57%, respectively, but decreased the relative abundance of the dominant fungal phylum “Chordata” and “Streptophyta” by 11 and 20%, respectively, in the inter-root soil of kiwifruit, and had a significant impact on the microbial (bacteria and fungi) diversity. In addition, inter-cropping could greatly increase the inter-root soil carbon sequestration (*PccA, korA/B/C/D, fhs*, and *rbcl/s*), carbon degradation (*abfD*), organic nitrogen mineralization (*GDH2*), denitrification (*napA/B, nirB, norB*), organic phosphorus mineralization (*phop, phn*), and inorganic phosphorus solubilization (*gcd, ppk*) gene abundance. The gene co-occurrence network indicated that soil *korB, nirB*, and gnd key functional genes for carbon, nitrogen, and phosphorus cycling in kiwifruit inter-root soils and their expression was up-regulated in the inter-cropping group. Structural equation (SEM) further showed that soil total nitrogen, organic matter, total carbon and acid phosphatase had significant effects on microbial diversity (*p* < 0.05) and soil carbon cycling gene korB and phosphorus cycling gene purH (*p* < 0.001), while korB and purH had positive effects on kiwifruit quality. In conclusion, intercropping perilla in kiwifruit orchards changed the structure of bacterial and fungal communities in the inter-root soil of kiwifruit, but I believe that intercropping perilla stimulates carbon degradation, leading to carbon emission and serious loss of soil nutrients, and that prolonged intercropping may adversely affect the quality of kiwifruit, and thus its limitations should be noted in future studies.

## 1 Introduction

Inter-cropping is an agricultural practice in which two or more crops are grown simultaneously on the same land during the same growing period (Zhou et al., 2011). Inter-cropping systems allow efficient plant access to nutrients; suppression of weeds and pests; improvement of soil microbial community and resource utilization of unused vacant land (Mousavi and Eskandari, 2011). In inter-cropping systems, cover crops alter soil properties by inputting plant residues and root secretions, which affect microbial communities and enzyme activities (Wang et al., 2016), while providing more organic substrates for microbes (Liu et al., 2013). Soil microorganisms play a key role in participating in nutrient cycling (Sun et al., 2015), and changes in microbial communities affect soil nutrient uptake and transformation mainly through phytophagous root secretions (Beckers et al., 2017). In soil ecosystems, soil microorganisms are also important decomposers that can break down organic macromolecules into organic small molecules for plant uptake through the secretion of soil extracellular enzymes (e.g., leucine aminopeptidases, cellobiose hydrolases, acid phosphatases). Inter-cropping is a traditional cropping system with great potential for sustainable agricultural development (Knörzer et al., 2009). It has been shown that sugarcane-peanut inter-cropping increased soil bacterial abundance and diversity (Pang et al., 2022), and cover crops were also found to increase soil bacterial diversity and species abundance in subtropical orchards (Cui et al., 2015). Inter-cropping systems improved soil nutrients, enzyme activities, and microbial diversity in apple orchards (Qian et al., 2015), and similarly, sugarcane-legume inter-cropping significantly increased effective potassium, total phosphorus, and soil enzyme content of soils (Solanki et al., 2019).

Perilla [*Perilla frutescens* (L.) Britt] is an annual herb in the genus perilla of the Labiatae family (Peiretti, 2011). Perilla seed oil can contain up to 65% α-linolenic acid, which is one of the highest α-linolenic acid content in terrestrial plants (Asif, 2011). Perilla leaves and stalks contain flavonoids, rosmarinic acid, volatile oils and other chemical compounds (Yamazak et al., 2008), and the fresh leaves of perilla are popular as a vegetable in China, Korea, Japan and other countries (Ito et al., 2008). The nutritional and functional characteristics of perilla are important for its development in the fields of medicine, industry, and food (Sa et al., 2013). ‘Guichang’ kiwifruit (Actinidia deliciosacv. Guichang) is one of the specialty boutique fruits in Guizhou Province and an important pillar of the local industry, with a planting area of up to more than 13,333 hectare in Guizhou Province at present. With the increasing agricultural production, the use of large amounts of chemical fertilizers and pesticides has resulted in the decline of soil quality, low nutrient utilization and negative impacts on human health (Savci, 2012; Yang et al., 2018; Chen et al., 2021). Inter-cropping, green manure, application of animal manure, and nano-agriculture are widely used to improve the ecological functions of cropping systems (Wezel et al., 2014; Behl et al., 2022). In China, inter-cropping systems play a key role in ensuring food and nutrient availability and increasing farm income (Ahoyo et al., 2007; Hao et al., 2021). Inter-cropping is recognized as one of the global sustainable agricultural practices because of its advantages such as improved soil quality and crop yield (Tang et al., 2021).

Most previous studies used 16S rRNA gene library construction and denaturing gradient gel electrophoresis (DGGE) methods to characterize soil microorganisms, but only a small number of dominant soil microbial taxa could be determined (Chen et al., 2012; Xi et al., 2019), and with the rapid development of DNA sequencing technology, the macro-genome sequencing technology can more comprehensively determine the soil microbial composition and function of each taxon (Navarrete et al., 2015). Many studies have shown that inter-cropping with legumes can reduce nitrogen input and improve crop yields as well as land use efficiency (Hu et al., 2020; Liu et al., 2021). However, previous studies have focused on soil fertility, crop yield, and the distribution of a few dominant microorganisms, yet only a few genes, such as *korB*, *amoA*, *appA*, *phn*, and *phoU*, have been identified in soil so far (Lagos et al., 2016; Dai et al., 2020). Little is known about the structure of inter-root soil microbial communities and functional genes for carbon, nitrogen and phosphorus cycling under inter-cropping of Labiatae aromatic plants. Therefore, this study utilizes macro-genomic technology to deeply excavate key functional genes in the inter-root soil of intercropped crops, which is of great significance for improving carbon, nitrogen and phosphorus cycling in ecological agricultural systems.

In this study, an inter-cropping pattern with different perilla varieties and planting densities was set up under the kiwifruit forest to compare with monocropping kiwifruit. Changes in microbial community and carbon, nitrogen and phosphorus cycling genes in kiwifruit inter-cropping soil were analyzed by macro gene sequencing, and the effects of kiwifruit-perilla inter-cropping on nutrient factors, enzymes, microbial community structure and nutrient cycling genes in kiwifruit inter-rooting soil were studied. This study provides a theoretical basis for the development of inter-cropping systems and sustainable agriculture, and is of practical significance for exploring suitable planting patterns under forests and improving land utilization.

## 2 Materials and methods

### 2.1 Experimental design

The experiment was set up in the experimental base of Zhen Qiyi Fruit Tree Research Institute, Xiuwen County, Guizhou Province, China. The average annual temperature is 16°C, the altitude is 1,300 meters above sea level, the climate was subtropical monsoon humid zone, and the average annual precipitation was between 1,000 mm and 1,250 mm. The 6-year-old ‘Guichang’ kiwifruit was used as the test material and cultivated in a “T” frame. Perilla seeds provided by Guizhou Rapeseed Institute, and perilla seedlings were planted in April 2022 and transplanted at the end of May. Soil physical and chemical properties before intercropping: organic matter 43.93 g/kg, pH 5.5, total carbon 21.06 g/kg, total nitrogen 2.43 g/kg, total phosphorus 10.91 g/kg, total potassium 1.45 g/100 g, alkaline nitrogen 217.67 mg/kg, available phosphorus 1935.75 mg/kg, available potassium 294 mg/kg.

**Figure 1:**
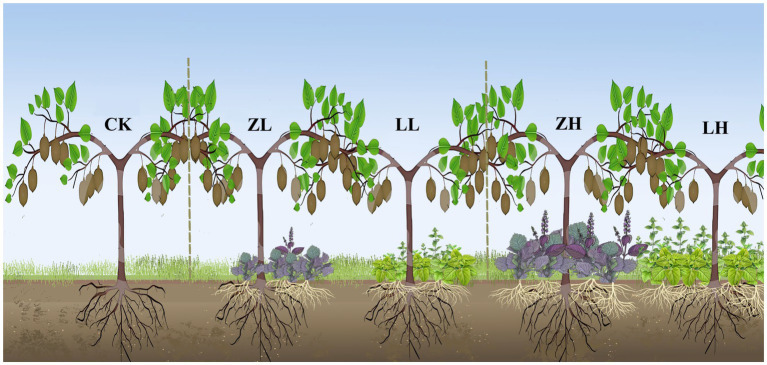
Inter-cropping mode of kiwifruit and perilla.

The experiment was set up as four inter-cropping patterns with two varieties of *P. frutescens* (Qi Su 2, with leaves positively green with deep purple backs for perilla variant, and Gui Zi 2, with purple on both sides for Hui Hui Su) and two planting densities of *P. frutescens* (Row spacing: 50 cm x 17 cm, equivalent density 75,000 plants/hm^2^; Row spacing: 50 cm × 27 cm, equivalent density 112,500 plants/hm^2^), namely: Qi Su 2 + 112,500 plants/hm^2^ (LH), Gui Zi 2 + 112,500 plants/hm^2^ (ZH), Qi Su 2 + 75,000 plants/hm^2^ (LL) and Gui Zi 2 + 75,000 plants/hm^2^ (ZL), and kiwifruit monoculture treatment (CK) as the control (Figure 1). The experiment was conducted in a randomized block design with 36 m^2^ (3 m × 12 m) per plot, three biological replications, and a total of 15 plots, 4 kiwi trees per plot. At the end of May 2022, perilla was transplanted to the kiwifruit rows (perilla and kiwi trees spaced 50 cm apart in rows), and since no chemical fertilizer was added to this kiwifruit orchard for a long time, urea (150 kg/hm2) was applied once to 15 plots 15 days after the transplanting of perilla for better survival of the perilla seedlings, and no fertilizer was applied thereafter.

### 2.2 Soil sample collection

At the beginning of October 2022 (perilla harvesting period), three kiwifruit plants of similar size were selected in each plot according to the equidistant sampling method, and the fine roots were dug with a sterilized spade at a depth of 0–20 cm around the kiwifruit main roots, and the inter-root soil adhering to the fine roots was shivered into self-sealing bags, and the inter-root soil of the three kiwifruit plants in each plot was fully mixed to make a total of 15 soil samples, where one portion of collected soil sample was freeze in liquid nitrogen, and brought back to the laboratory to be preserved in refrigerator at-80C for soil macrogenomic assay, while other portion of soil sample was used for the determination of soil physiochemical properties and enzyme activities after air drying.

### 2.3 Determination of soil physicochemical properties and enzyme activities

Reference for the determination of soil physical and chemical properties (Bao, 2000),Soil pH was determined by glass electrode method; soil organic matter (SOM) content was determined by potassium dichromate oxidation-external heating method; soil total carbon (TC) content was determined by TOC analyzer using tinfoil embedding; soil alkaline nitrogen (AN) content was determined by the alkaline diffusion method through NaOH hydrolysis; soil available potassium (AK) content was determined by leaching using NH4OAc and then by flame photometry; Soil available phosphorus was extracted by leaching with sodium fluoride hydrochloric acid, and soil available phosphorus content (AP) was determined by molybdenum antimony antimony colorimetric method. Total phosphorus (TP) in soil samples was determined by HClO4 digestion and phosphorus in extracts was determined spectrophotometrically; total nitrogen (TN) in soil was determined by Kjeldahl method using sulfuric acid-accelerant digestion; and total potassium (TK) in soil was determined by NaOH alkali fusion and flame photometry.

Soil enzyme assay reference (DeForest, 2009). Soil leucine aminopeptidase (S-LAP) calculated S-LAP activity by catabolizing L-leucyl-p-nitroanilide to produce p-nitroanilide and measured its absorbance at 405 nm (Multi-function Enzyme Labeler,Scientific Fluoroskan Ascent FL,Thermo); soil β-Xylosidase (S-β-XYS) calculated S-β-XYS activity by catalyzing the production of p-nitrophenol from p-nitrophenol-β-D-glucopyranoside, and determining the rate of increase of p-nitrophenol’s light absorption at 405 nm. soil β-glucosidase (S-β-GC) catalyzes the production of p-nitrophenol from p-nitrophenyl-β-D-glucopyranoside, and the characteristic light absorption was measured at 405 nm to calculate the activity of S-β-GC; acid phosphatase (S-ACP) catalyzes the hydrolysis of disodium benzoate phosphate to produce phenol and disodium phosphate, and the amount of phenol produced was measured to calculate the S-ACP activity; cellobiose hydrolase (S-CBH) catalyzes the formation of p-nitrophenyl-β-D-cellobiose to produce p-nitrophenol (PNP), which has a characteristic light absorption at 405 nm to obtain the magnitude of S-CBH activity; soil N-acetyl-β-D-aminoglucosidase (S-NAG) generates p-nitrophenol by catabolizing N-acetyl-β-D-glucoside, and the rate of light absorption was measured at 405 nm to calculate S-NAG activity. The specific steps were referred to the instruction manual of the kit (Suzhou Dream Rhinoceros Biomedical Technology Co., Ltd.). Three parallel samples were set for each sample, and the average value of the parallel samples was used as the final data of the experiment to meet the requirements of precision and accuracy of the test results.

### 2.4 Soil microbial community macro-genome determination

Macrogenomics (next-generation sequencing) BGI sequencing was performed on all 15 soil samples (metabarcoding sequencing). The steps were as follows: 0.25 g of soil sample was weighed accurately and DNA was extracted from the soil samples using the DNeasy® PowerSoil® Kit. 1% agarose gel electrophoresis was used to check the integrity and purity of the extracted DNA, and then Qubit 4.0 was used to accurately quantify the DNA concentration. Input DNA was fragmented by mechanical interruption (ultrasound), while the fragmented DNA ends were flattened and phosphorylated at the 5′ end and the dA tail was added at the 3′ end. The ends of the fragmentation end-remediation products were subsequently linked to sequencing junctions (AdapterLigation) and purified by the magnetic bead method. For libraries with adapters, library fragments were screened with two rounds of VAHTS DNA Clean Beads (0.56×, 0.2×), and then the DNA libraries were amplified by PCR (NEBNext®Ultra™ DNA Library Prep Kit for Illumina®), and after the library construction was completed, the available concentration of the library (3 nM) was accurately quantified by qPCR. Finally, sequencing was performed on the Illumina platform PE150.

Firstly, low quality sequences were removed from the raw data using fastp software to get 1,280,849,442 clean reads, Clean data 190,845,503,376 bp. The average number of contigs per sample after assembly was 478,550, and the average genome size was 415,691,776 bp. The non-redundant gene set obtained after gene prediction and de-redundancy contained 3,853,915 genes with a total size of 1,796,397,822 bp. Sequences were aligned to the reference genome using bowyie2, and un-aligned bam files were extracted using Samtools (Danecek et al., 2021) (− f 4 parameter) after extracting the unmatched bam files, bam2fastx tool was used to convert to get the fq sequences, and genome assembly analysis was performed for each sample using MEGAHIT (Li et al., 2015) software, after assembly each sample yielded an average of 478,550 contigs, and an average genome size of 415,691, 776 bp. The non-redundant gene set obtained after gene prediction and de-redundancy contained 3,853,91 genes with a total size of 1,796,397,822 bp. ORF prediction of the assembly results was carried out by calling Prodigal through Prokka (Seemann, 2014) software, and after gene prediction, the genome assembly results were analyzed by using CD-HIT (Huang et al., 2010) to remove redundancy from the annotated genes, and select the longer ones in each class as UniqueGene, with the similarity threshold set to 95% and the coverage threshold set to 90%. Based on the BLAST (Camacho et al., 2009) software comparison method, the protein sequences of the predicted genes were compared with the KEGG and GO functional databases, and the results of gene function annotation were obtained.

### 2.5 Statistical analysis

Species annotation at each level was done using Kraken2 (Wood et al., 2019) + Bracken; differential expression analysis was mainly done using the R language package clusterProfiler. α-diversity indices were calculated using USEARCH software; Raw data collected from the experiment was arranged and calculated using Microsoft Excel; One way ANOVA was performed using SPSS 26 to calculate the mean differences among the treatments; Origin 2021 was used to draw histograms of relative abundance of soil enzymes and soil microbial communities; pheatmap(R) was used to draw a heatmap of the correlation between environmental factors and carbon, nitrogen, and phosphorus cycling genes; and Gephi was used to draw a network map of the cycling genes; in order to further study the effects of environmental factors on the quality and quality of carbon, nitrogen, and phosphorus cycling genes and kiwifruit, the following steps were taken. Genes and kiwifruit quality, structural equation models were constructed using Amos 26 according to the maximum likelihood method.

## 3 Results

### 3.1 Effects of different cropping patterns on the physicochemical properties of kiwifruit inter-root soils

**Figure 2:**
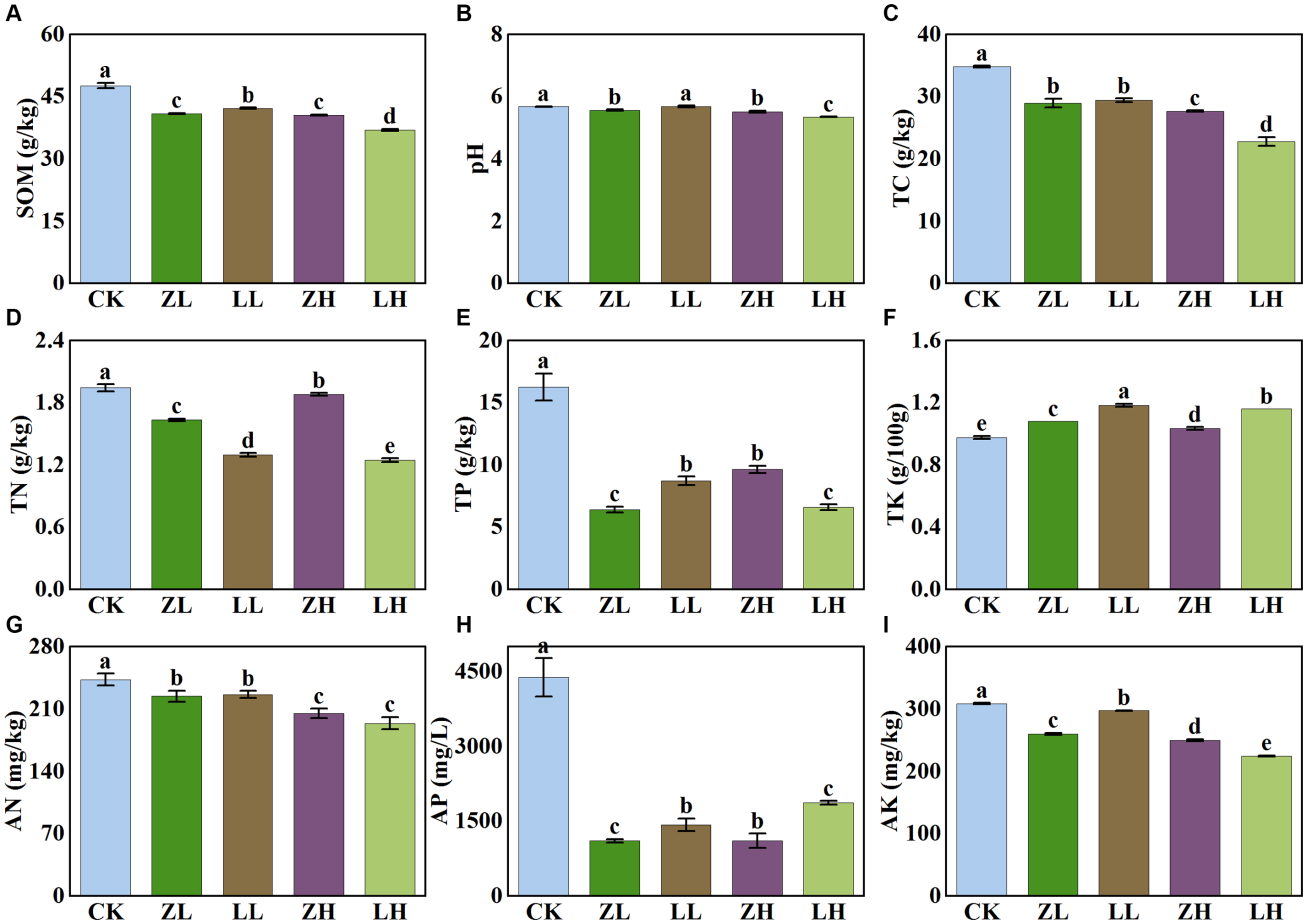
Physico-chemical properties of soil in different treatments. Different lowercase letters indicate significant differences (*p*< 0.05). **(A)** SOM,soil organic matter; **(B)** soil pH; **(C)** TC, total carbon; **(D)** TN, total nitrogen; **(E)** TP, total phosphorus; **(F)** TK, total potassium; **(G)** AN, alkaline nitrogen; **(H)** AP, available phosphorus; **(I)** AK, available potassium.

There were significant differences in soil physicochemical properties under different inter-cropping patterns (Figure 2). Compared with CK, all physicochemical indexes decreased in the inter-cropping group except TK. Soil pH under different treatments showed a slight variation in their values (5.35–5.68); soil SOM and TC contents showed the same trend and were the lowest under LH and ZH treatments; soil TN, AN, TP, and AK were all the lowest under LH treatment; for AP, they all decreased significantly in the inter-cropping group, with the lowest contents in ZL and ZH. Compared with CK, TC content was significantly decreased under the intercropping treatments. The results showed that inter-cropping treatments had less effect on kiwifruit inter-root soil pH and AN, but more effect on soil TP and AP, and with the change of different planting methods, it would have some effect on soil TN and AN.

### 3.2 Effects of different cropping patterns on the enzyme activities of kiwifruit inter-root soil

**Figure 3:**
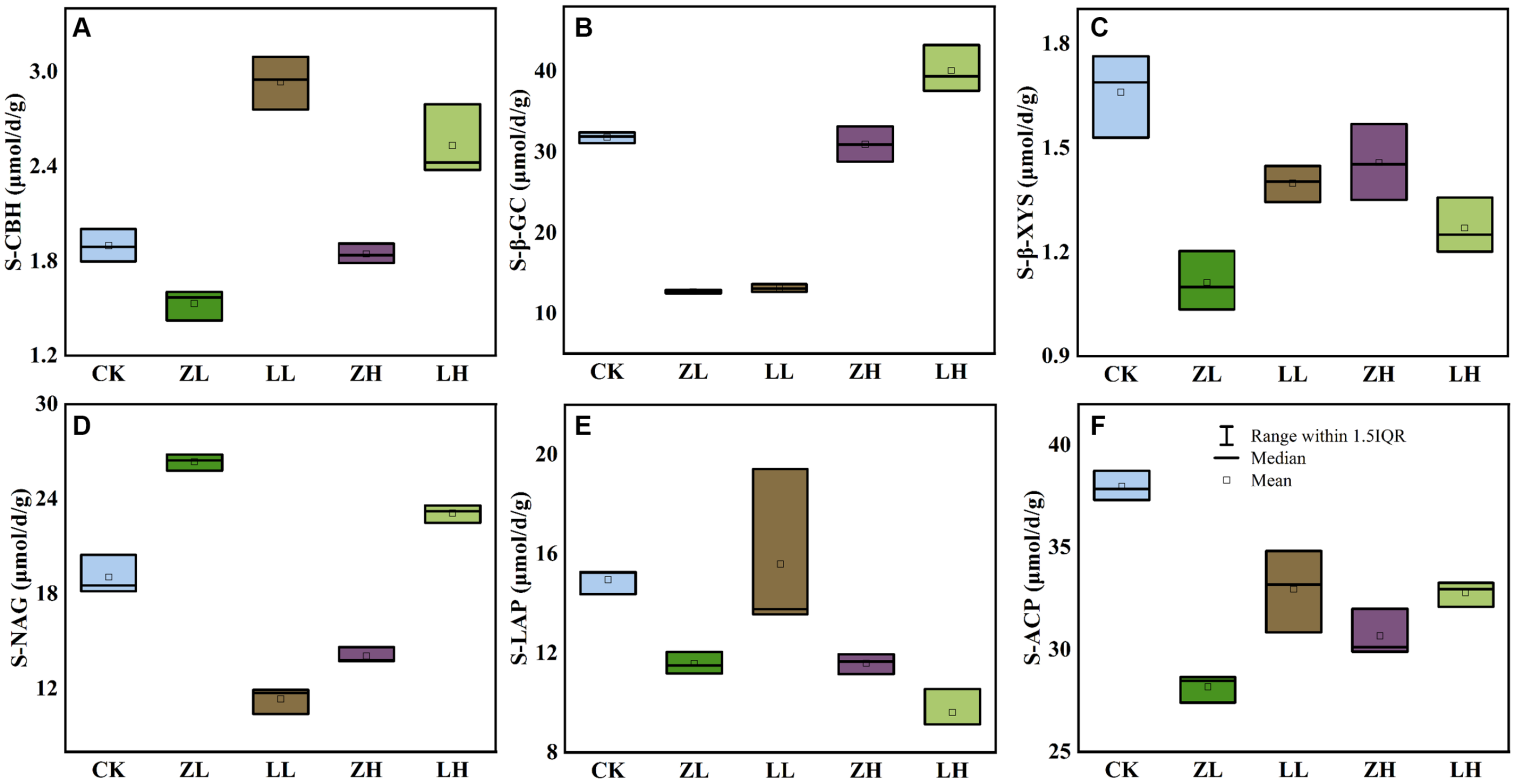
Effect of planting mode on soil enzyme activity. **(A)** S-CBH, soil cellulose disaccharide hydrolase; **(B)** S-β-GC, soil β-Glucosidase; **(C)** S-β-XYS, soil β-Xylosidase; **(D)** S-NAG, Soil N-acetyl-β-D-glucosidase; **(E)** S-LAP, soil leucine aminopeptidase; **(F)** S-ACP, soil acid phosphatase.

Six soil extracellular enzymes were mainly involved in the transformation of soil carbon, nitrogen and phosphorus data showed (Figure 3). For soil S-CBH (Figure 3A) and S-β-GC (Figure 3B) were significantly higher than CK in the LH group, indicating that increasing planting density increased and plant variety changes would promote these two enzyme activities, but both intercropping groups decreased S- β-XYS (Figure 3C) content; for S-NAG (Figure 3D), ZL and LH were the highest, compared to CK increased by 38.31 and 21.30%, while LL and ZH decreased 67.84 and 35.59, respectively, compared with CK, and it was also found that LL and LH were higher than ZL and ZH, which indicated that S-NAG responded sensitively to changes in planting density and variety; for soil S-LAP (Figure 3E) and S-ACP (Figure 3F), ZL, LL, ZH and LH were lower than CK, where the activity of S-LAP decreased with increasing planting density of Qi Su2, S-ACP was highest in CK, and ZH was higher than ZL, indicating that S-ACP increased with increasing planting density of Gui Zi2. The above results indicated that the enzyme activity would be changed with the change of planting method.

### 3.3 Species composition of soil microbial communities under different cropping patterns

**Figure 4:**
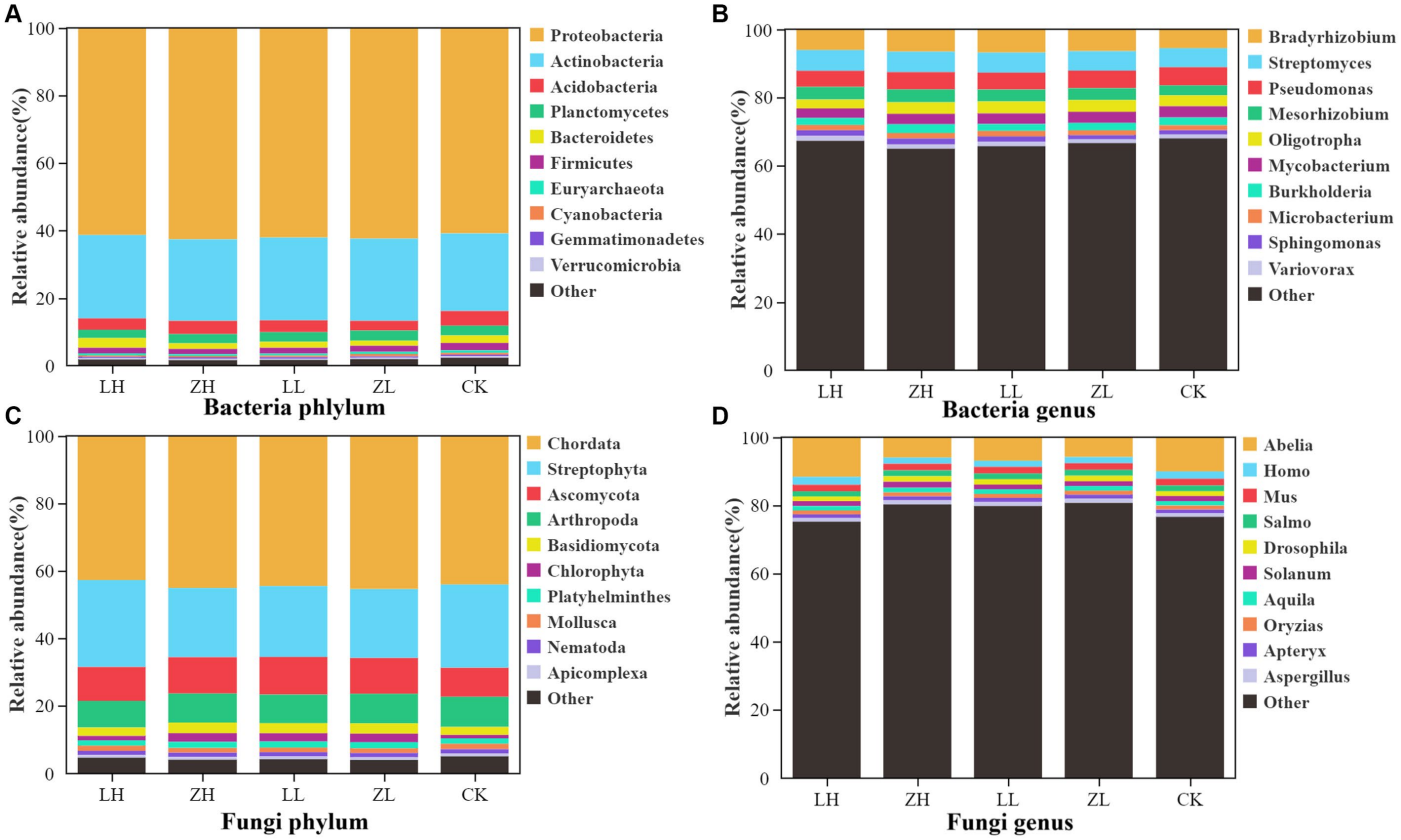
Relative abundance of the main taxa of bacteria **(A)** and fungi **(C)** at the phylum level, and the relative abundance of the main taxa of bacteria **(B)** and fungi **(D)** at the genus level.

The main dominant bacterial phyla of kiwifruit inter-root soil were Proteobacteria, Actinobacteria and Acidobacteria (Figure 4A), and the intercropping conditions greatly enhanced the number of soil Proteobacteria and Actinobacteria, with the highest abundance in the LH group; the dominant bacterial genera of inter-root soil were Bradyrhizobium, Streptomyces and Pseudomonas (Figure 4B), and the relative abundance of Bradyrhizobium, Streptomyces and Pseudomonas was generally enhanced in the intercropping group under intercropping conditions; the dominant fungal phyla were Chordata and Streptophyta (Figure 4C), except for the LH group, the intercropping groups generally decreased the relative abundance of the dominant fungal phyla Chordata and Streptophyta; the dominant fungal genera were Abelia, Homo and Mus (Figure 4D), except for the LH group, the intercropping groups generally decreased the relative abundance. The results showed that intercropping treatments generally increased the bacterial abundance and decreased the fungal abundance.

### 3.4 Analysis of soil microbial diversity under different cropping patterns

**Figure 5:**
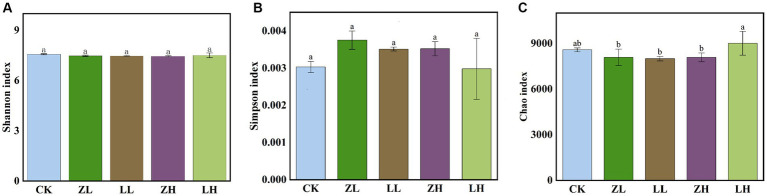
shannon_e index **(A)** rhizosphere microbial relative abundance, Simpson index **(B)** and Chao1 index rhizosphere microbial richness index **(C)**. Different lowercase letters indicate significant differences (*p* < 0.05).

The shannon_e (Figure 5A) and Simpson (Figure 5B) indices were not significantly different across treatments, while the chao1 (Figure 5C) index was not significantly different in LH and CK and was the highest among all treatments. Non-MetricMulti-Dimensional Scaling (NMDS) was used to support the reliability of the data in this experiment by side-by-side validation of the overall changes in the structure of soil microbial (bacterial and fungal) communities in kiwifruit inter-roots under inter-cropping treatments (Supplementary Figure S1). Different perilla cropping patterns significantly altered the microbial community structure changes in kiwifruit inter-root soil. After intercropping perilla, the microbial communities of the soil were mainly located in the second, third and fourth quadrants, implying that these orders are likely associated with intercropping, whereas CK treatment was located in the first quadrant indicating a clear association with monocropping, with ZL being the closest to Ck, LH being the furthest away from CK, and the smallest within-group differences in ZH and LL. Thus, all planting patterns had significant effects on the kiwifruit inter-root soil microbial community (STRESS <0.2).

### 3.5 Abundance of functional genes for soil carbon, nitrogen, and phosphorus cycling

Macro-genome sequencing analysis revealed 218, 42 and 94 genes related to C, N and P cycling in kiwifruit inter-root soils, respectively. PcoA showed that C, N and P cycling related genes differed significantly between treatments, suggesting that inter-cropping of perilla altered the C, N and P cycling (Supplementary Figure S2).

**Figure 6:**
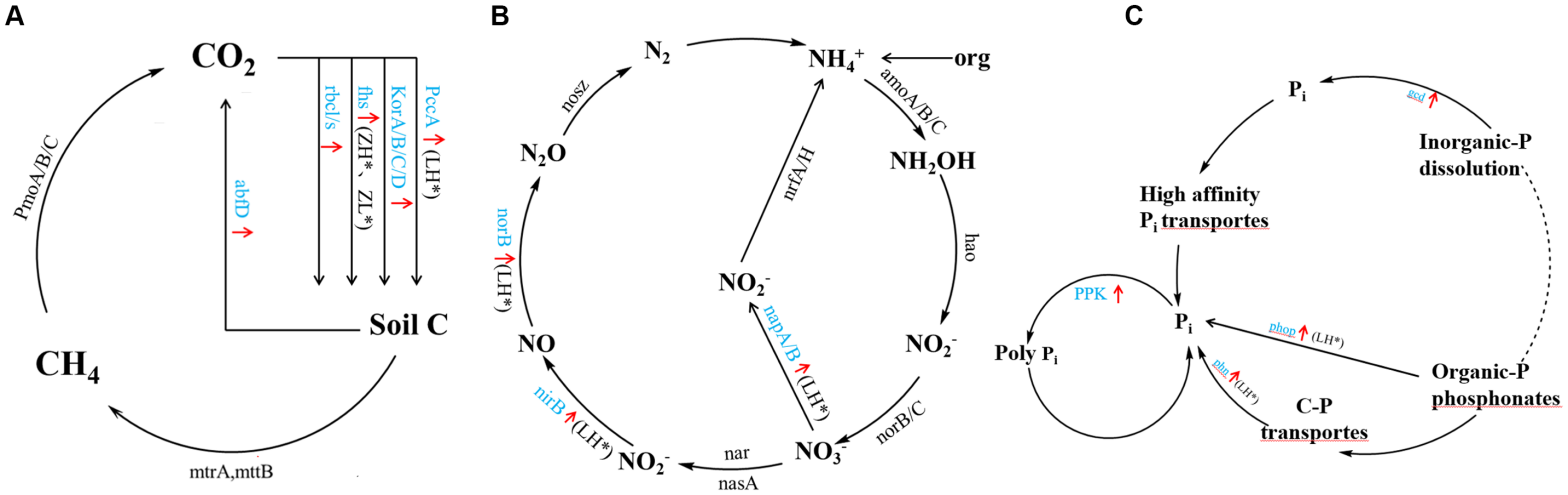
A diagram of carbon **(A)**, nitrogen **(B)**, and phosphorus **(C)** cycling processes based on metagenomic sequencing. Black font indicates no significant Black font indicates no significant difference between inter-cropping and monocropping, blue font and red arrow indicate an increase in all inter-cropping groups, and * (*p* < 0.05) indicates a significant increase.

Inter-cropping perilla also generally increased genes involved in carbon, nitrogen, and phosphorus cycling in kiwifruit inter-root soils compared to CK (Figure 6), including carbon sequestration (*PccA, korA/B/C/D, fhs, and rbcl/s*), carbon degradation (*abfD*) (Figure 6A), organic nitrogen mineralization (*GDH2*), denitrification (*napA/B, nirB, norB*) (Figure 6B), organic phosphorus mineralization (*phop* and *phn*), inorganic phosphorus dissolution (*gcd, ppk*) (Figure 6C). Among them, the LH group significantly (*p* < 0.05) increased *PccA* gene abundance, the ZH and ZL groups significantly increased *fhs* and *mttB* gene abundance, and the LH group significantly (*p* < 0.05) increased *napA/B, nirB, norB* and *phop, phn* and *ppk g*ene abundance. Compared with CK, the inter-cropping group had no significant effect on methane metabolism (*PmoA/B/C*), nitrification (*amoA/B/C, hao* and *norB/C*), and nitrogen fixation (*nrfA/H*), and there were no significant differences between the different perilla planting patterns.

### 3.6 Expression and co-occurrence network analysis of functional genes for soil carbon, nitrogen, and phosphorus cycling

**Figure 7:**
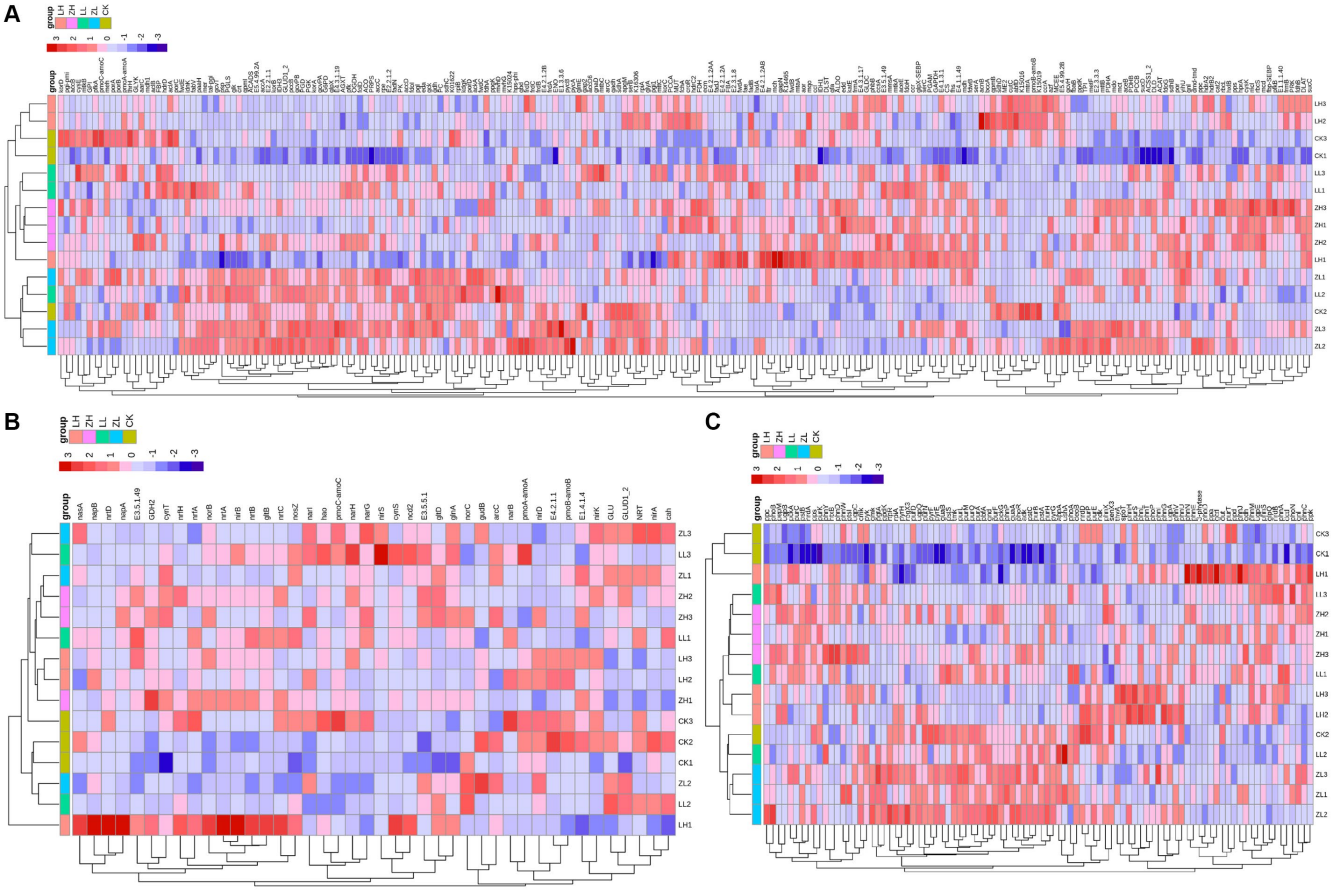
Represents the expression and relationship of carbon **(A)**, nitrogen **(B)**, and phosphorus **(C)** cycling genes with different treatments in the rhizosphere soil of kiwifruit.

Based on the gene expression profiles of carbon, nitrogen and phosphorus cycles (Figure 7), for carbon cycle (Figure 7A), the largest relative abundance was *ACAT*, *paaH* and *CS* accounting for 2.78, 2.57 and 2.46% of the total carbon cycle genes, respectively, and the three genes in the inter-cropping group were larger than those in the control group; for nitrogen cycle (Figure 7B), the largest relative abundance was *glna*, *GLUD1_2* and *cynT*, accounting for 26.93, 11.92 and 2.46%, respectively, and all of them were higher than the gene abundance in the inter-cropping group than in the control group; for the soil phosphorus cycle (Figure 7C), the top three abundances were *nrdA*, *gcd* and *pstS*, accounting for 4.44, 3.65 and 3.57%, and all of them were higher than that in kiwifruit monoculture. From Figure 7, it can be found that most of the carbon, nitrogen and phosphorus cycling genes were positively correlated with the samples of the inter-cropping group, and negatively correlated with CK, which side by side confirms that inter-cropping perilla generally increased the abundance of carbon, nitrogen and phosphorus cycling genes in the inter-root soil of kiwifruit.

**Figure 8:**
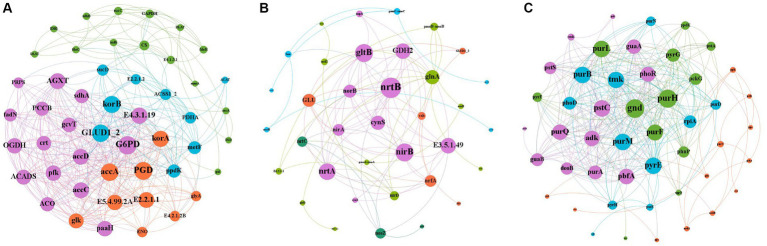
Analysis of the Gene Interaction Network of Carbon **(A)**, Nitrogen **(B)**, and Phosphorus **(C)** Cycling in the Rhizosphere Soil of Kiwifruit. The size of each node is proportional to the number of connections.

Gene interactions co-occurrence network analysis (Figure 8), was used to examine the co-occurrence patterns of carbon, nitrogen and phosphorus cycling genes in kiwifruit inter-root soil under five treatments. Overall, the carbon, nitrogen and phosphorus cycling genes were divided into four different modules, and there were 395 links and 49 nodes in the carbon cycling gene network, with *G6PD*, *GLUD1_2* and *korB* nodes located in the center of the network being the largest, and all of them were higher in inter-cropping than in the control group (Figure 8A). There were 89 links and 36 nodes in the nitrogen cycle gene network, Among them, *nrtB*, *nirB* and *gltB* nodes were the largest, and their gene abundance was significantly (*p* < 0.05) higher in the LH and ZH groups than in CK (Figure 8B). There were 316 links and 48 nodes in the phosphorus cycling gene network, among which *purH*, *gnd* and *purM* nodes were the largest, and the relative abundance of all three phosphorus cycling genes was the highest in the LH group and significantly (*p* < 0.05) higher than that in the CK group (Figure 8C).

### 3.7 Correlation analysis of soil carbon, nitrogen, and phosphorus cycling functional genes with environmental factors and functional microorganisms

**Figure 9:**
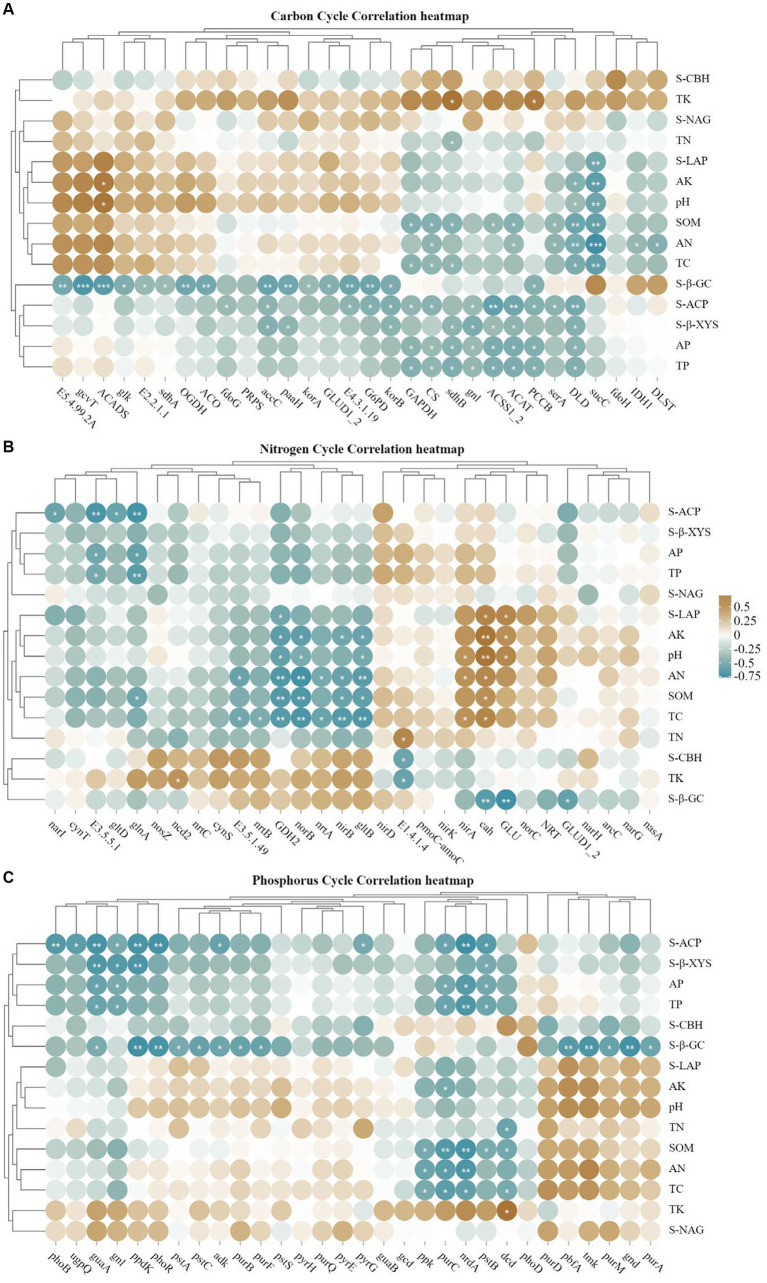
**(A–C)** Represents the correlation analysis between soil carbon, nitrogen and phosphorus cycling genes (top 30), soil physicochemical properties and enzyme activities (*p < 0.05; **p < 0.01).

Heat map analysis of soil carbon, nitrogen and phosphorus cycling genes correlated with soil physicochemical properties and enzyme activities (Figure 9), *korA/B*, which is associated with carbon fixation, was positively correlated with S-β-GC, and TC and SOM had high concordance, and negatively correlated with most of the carbon cycling genes (Figure 9A); *GDH2*, *norB* were highly significant negatively correlated with TC, SOM, AN and S-LAP, *glnA, nirB* and *gltB* were all significantly negatively correlated with SOM, where *gltB* and AN were highly significantly negatively correlated, and *nirA* was the opposite, but TN showed a weak correlation with the nitrogen cycle; for the soil phosphorus cycle (Figure 9C), most of the genes were negatively correlated with AP, TP and S-ACP.

### 3.8 Analysis of plant growth

**Figure 10:**
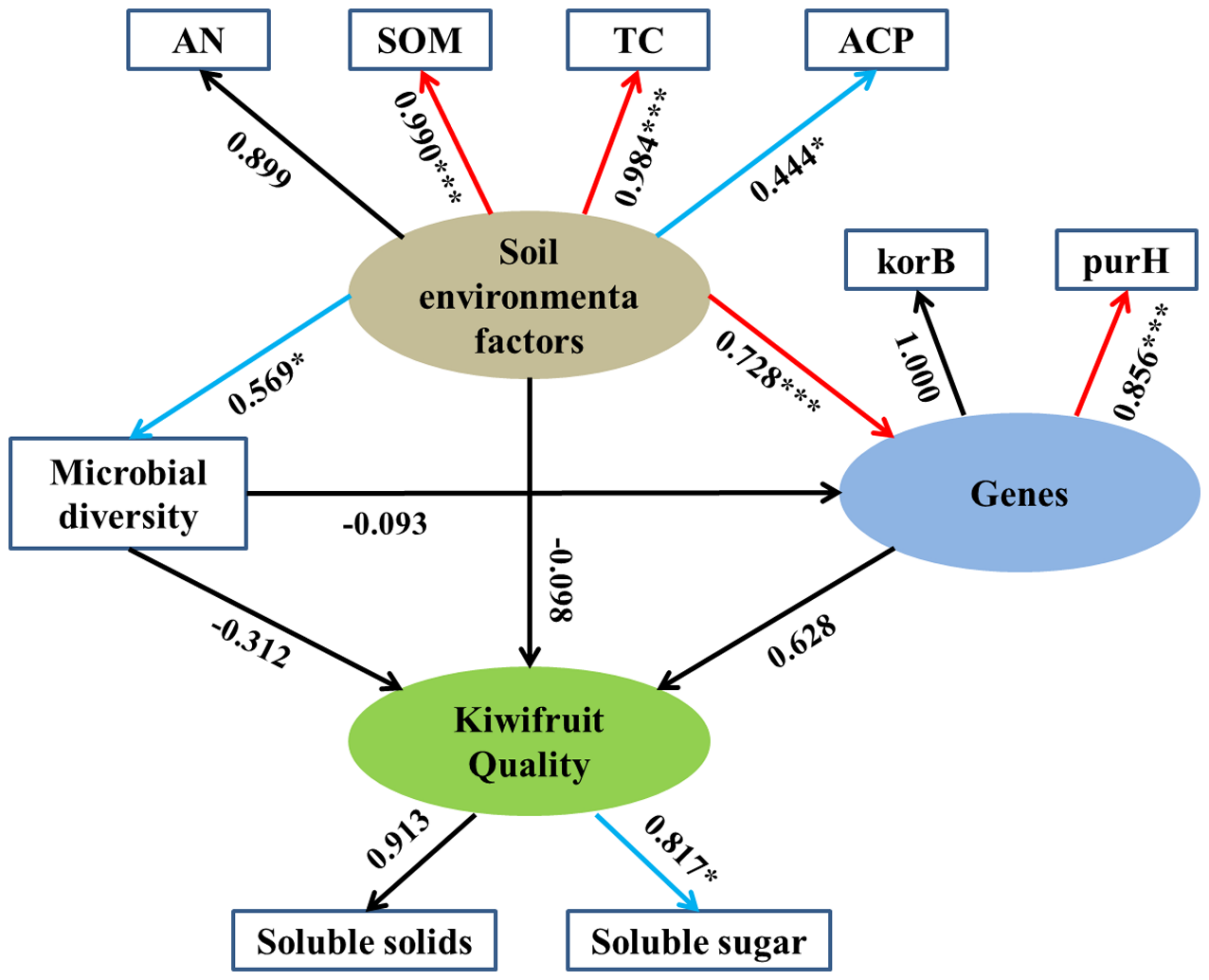
Structural equation modeling (SEM) was used to analyze the relationships between rhizosphere soil physicochemical properties, enzyme activities, microbial diversity, soil carbon and phosphorus cycling genes, and kiwifruit quality. Numbers next to arrows represent path coefficients (*p < 0.05; **p < 0.01; ***p < 0.001). Red arrows indicate strong correlations, blue arrows indicate weak correlations, and black arrows indicate insignificant correlations.

The results of SEM showed the direct and indirect effects of environmental factors on kiwifruit quality (Figure 10), where the latent variable “soil environmental factors” had a significant loading factor with SOM (0.990), TC (0.984) and ACP (0.444), except AN which showed a non-significant correlation. Soil environmental factors had a significant positive effect on soil carbon (korB) and phosphorus (purH) cycling genes with standardized path coefficients of 0.728 (*p* < 0.001) and but having weaker positive effect on soil microbial diversity with standardized path coefficients of 0.569 (*p* < 0.05). The latent variables “soil carbon and phosphorus genes” showed a non-significant correlation (0.628), whereas soil microbial diversity showed a non-significant negative correlation (−0.312) with kiwifruit quality. Meanwhile, the correlation between “soil environmental factors” and kiwifruit quality was not significant (−0.098), and the correlation between “soil microbial diversity” and soil carbon and phosphorus cycling genes was not significant (−0.093).

## 4 Discussion

### 4.1 Changes in soil nutrients and enzymes activities

Inter-cropping can increase soil nutrient content compared to monocropping (Xie et al., 2022). The results of the present study showed that inter-cropping perilla decreased AP and SOM, which was contrary to the results of the study that inter-cropping maize-cotton could increase soil effective phosphorus and organic matter (Zhao et al., 2011), but increased soil TK and decreased AK content, which indicated that the available potassium had been transformed or incorporated into soil minerals, thus increasing the total soil potassium content. Were both decreased, probably due to the large amount of N uptake by perilla leading to the decrease of TN and AN, which was consistent with the results of sugarcane-soybean inter-cropping (Yu et al., 2021). The decrease in soil TC and SOM contents under intercropping conditions compared with CK may be due to the fact that intercropping of perilla led to over-tillage of the land, resulting in the decomposition of organic matter in the soil at a faster rate than its accumulation, thus reducing the SOM content; it may also be due to the fact that intercropping resulted in the exposure of the soil to the external environment for a long period of time, and the relative seriousness of erosion, which led to the loss of soil SOM content, thus reducing the TC content. It has been shown that inter-cropping can reduce soil pH through the release of hydrogen ions from root secretions (Sun et al., 2020), which was reinforced by the present study. Decrease in soil AP and TP content due to intercropping of perilla is due to differences in crop phosphorus requirements, which vary from crop to crop, and perilla, a crop that consumes a large amount of phosphorus, and this competitive relationship may affect soil phosphorus content. For example, it has been shown that intercropping wheat with faba bean increased the inter-root quick phosphorus content of wheat during the period from nodulation to filling, and significantly reduced the inter-root quick phosphorus content and soil phosphorus content during the maturity period (Li et al., 2016).

It has been reported that inter-cropping can increase the functional potential of below-ground microbial communities by increasing plant diversity, and enzyme activities can be influenced by plant species (Kourtev et al., 2002). According to the reviewer’s opinion, S-ACP has been put to the back, and only soil nutrients are discussed in the first paragraph of 4.1, and only soil enzyme activities are discussed in the latter paragraph. S-ACP mainly regulates the effectiveness of soil P (Li et al., 2021), while S-ACP activity was generally reduced in the intercropping group, which may be due to the intercropping conditions that cause changes in soil microbial communities, soil nutrients, and inter-root secretion, which lead to changes in this enzyme’s stability and activity. This led to changes in the stability and activity of the enzyme, and in addition intercropping reduced soil phosphorus content and soil phosphorus supply capacity, which may be an important reason for the decrease in S-ACP content (Wang et al., 2022, 2023). Soil S-β-GC and S-CBH were the highest in the LH group compared to CK, a result that suggests that the increase in this enzyme may be due to differences between root complete and root incomplete barriers due to the effect of root secretions between intercropped roots (Kourtev et al., 2002). It has been shown that the promotion of enzyme activities is due to root secretions rather than the root itself (Xu et al., 2007; Hu et al., 2013; Li et al., 2018), and the present study more validates this result, and also suggests that reasonable inter-cropping can improve soil enzyme activities. However, the monosaccharides released by S-β-XYS activity were further used as a carbon source by soil microorganisms (Bosetto et al., 2016), which led to a decrease in this enzyme activity due to a decrease in TC content in the inter-cropping group compared to CK. NAG and S-LAP are enzymes involved in the soil nitrogen cycle, and in the present study, the NAG was higher than that of CK in ZL and LH, whereas it was lower than that of CK in LL and ZH. However, in all the inter-cropping groups TN and AN were decreased, indicating that S-NAG was not correlated with TN and AN, in agreement with the results of a previous study (Kracmarova et al., 2020), while S-LAP showed a positive correlation with AN.

### 4.2 Changes in carbon, nitrogen, and phosphorous cycling genes under perilla-kiwifruit inter-cropping

In this study, inter-cropping perilla significantly increased soil carbon fixation gene abundance (e.g.*, PccA*, *korA/B/C/D*, and *fhs*) compared with CK. The application of chemical nitrogen fertilizers helped to maintain a relatively stable level of soil carbon fixation similar to the results of a study on the abundance of degradation-related genes in agricultural soils (Yu et al., 2020), which may be due to the ability and rate of heterotrophic microorganisms to utilize the C source, i.e., a small amount of organic carbon is rapidly utilized by heterotrophic microorganisms thus leading to carbon fixation (Ge et al., 2013). In the present study SOM and TC were significantly decreased in the intercropping group as compared to CK. intercropping may increase the time the soil is exposed to the external environment, which increases the risk of soil erosion and water erosion. This would lead to loss of organic matter from the soil, thus reducing the total soil carbon content.in addition to the effects of SOM and TC, water content has a key effect on genes related to the carbon sequestration process (Quiza et al., 2014), and we hypothesized that inter-cropping can retain more water in the soil profile compared to traditional monocropping, thus corroborating the fact that some carbon cycling genes would be up-regulated in terms of their expression under inter-cropping treatments. Different environmental conditions, crop types, and fertilizer inputs affect the abundance of genes for soil carbon degradation (Huang et al., 2019; Jing et al., 2021). It has been shown that inter-cropping sugarcane with soybean (Yu et al., 2020) had a weak effect on soil carbon degradation genes in sugarcane, which significantly increased soil carbon degradation genes (*abfD*) with all inter-cropping groups in this study. This result was mainly attributed to the fact that microbial communities balance their elemental requirements through selective degradation under different external conditions (Hu et al., 2022), and since inter-cropping perilla consumes a large amount of organic compounds in order to maintain carbon stabilization, the promotion of carbon degradation genes abundance under inter-cropping conditions could be an important ecological mechanism for microorganisms to maintain elemental homeostasis in the soil (Luo et al., 2019), thus inter-cropping perilla accelerated the rate of carbon turnover in the region.

Nitrogen-degrading genes with high activity can contribute to the conversion of ammonia to *glnA* by NH4+ (*GDH2*) and the breakdown of ureA into ammonia, thereby accelerating the nitrogen cycle and providing more nutrients to plants, (Geisseler et al., 2010; Bernard and Habash, 2009). In this study both GDH2 and glnA were significantly elevated in the intercropping group. Kiwifruit and perilla intercropping led to changes in soil taxonomic and functional community structure and increased organic N mineralization (GDH2) in adjacent crops, which contrasted with the decrease in N mineralization due to sugarcane-soybean intercropping (Cong et al., 2015). However, *nirB* and *norB*, which are involved in the denitrification process, were significantly elevated only in the LH group, and it has been reported that the denitrification process may have accelerated nitrogen loss in the form of nitrogen gas (Kelly et al., 2021), resulting in the lowest levels of TN and AN in the LH. For DRNA (*napA/B*) was the highest in LH. *napA/B* is mainly responsible for the conversion of highly mobile NO3-to NH4+, which enhances nitrogen fixation to mitigate nitrogen loss (Vysotskaya et al., 2021). With the above results we hypothesized that excessive planting densities may contribute to nitrogen loss.

In our study, we found that the *gcd* gene, after *purH*, was the most abundant phosphorus cycling gene in kiwifruit inter-root soil, which is consistent with the results of other studies (Dai et al., 2020; Wu et al., 2021) that the *gcd* gene is a typical gene for inorganic phosphorus solubilization, and the expression of *gcd* was elevated in all inter-cropping groups. As a result of planting large amounts of perilla, its roots secrete large amounts of organic acids to accelerate the solubilization of inorganic phosphorus for its own uptake (Zhang et al., 2023). Similarly, *pstS* gene is the most abundant gene in kiwifruit inter-root soil. *pstS* is a high affinity phosphate-specific transport protein (Hsieh and Wanner, 2010), which is mainly involved in phosphorus uptake and transport system. It was found that *pstS* abundance was higher than CK in all inter-cropping groups. We hypothesized that *pstS* plays an important role in microbial phosphorus assimilation because phosphorus is highest in CK, and high phosphorus levels inhibit *pstS* transporter expression (Liu et al., 2023), suggesting that inter-cropping groups have more soluble phosphorus for plant uptake and utilization, while monocropping causes the accumulation of stable phosphorus, which is detrimental to crop uptake (Zhang et al., 2023). The co-occurrence network suggests that *gnd* gene may be a key functional gene in phosphorus cycling, and its main physiological function is to produce NADPH and participate in the response to abiotic stresses such as drought stress, oxidative stress, and osmotic stress. In this study, we found that all of these phosphorus cycling functional genes were greatly enhanced under inter-cropping treatment, and these genes could enable microorganisms to effectively utilize phosphorus and convert phosphorus into their biomass, promoting phosphorus cycling in the region.

### 4.3 Response of C, N, and P cycling to microbial taxa in symbiotic mode

In this study, bacteria were found to be dominant in kiwifruit inter-root soils, whereas fungi were not, which is consistent with the findings of Wu et al. (2021) Proteobacteria and Actinobacteria were generally increased in the inter-cropping group. For example, α-Actinobacteria and γ-Actinobacteria carrying *gcd* (Zhang et al., 2023). The Ascomycetes phylum has a high nutrient utilization capacity and is a major driver of functional changes in soil (Du et al., 2022). Microorganisms associated with carbon, nitrogen, and phosphorus have been reported to be similar at the phylum level (Du et al., 2023), suggesting that the dominant microbial taxa of Proteobacteria, Actinobacteria, and Acidobacteria can be recognized as species with multiple ecological functions (Crump et al., 2012; Sriswasdi et al., 2017; Yang et al., 2023). Therefore, in this study, the dominant microorganisms Proteobacteria and Actinobacteria can be considered as multifunctional taxa involved in carbon, nitrogen, and phosphorus cycling. Functional genes are closely related to the function of microorganisms in the soil carbon, nitrogen and phosphorus cycle, and therefore, functional genes have the potential to be important indicators of soil health and quality in agroecosystems.

## 5 Conclusion

Under inter-cropping kiwifruit with perilla, soil pH, SOM, TC, soil major nutrients (AN, TN, AP, TP, AK) as well as soil enzymes related to nitrogen and phosphorous were significantly decreased. Macrogenomics revealed that Proteobacteria, Actinobacteria and Acidobacteria play key roles in carbon, nitrogen and phosphorus cycling in kiwifruit inter-root soil. Our study also found that kiwifruit-purslane inter-cropping could promote carbon and phosphorus cycling, but excessive planting density caused nitrogen loss. Among them, soil *korB*, *nirB*, and *gnd* may be the key functional genes for carbon, nitrogen and phosphorus cycling in kiwifruit inter-root soil. In addition, structural equations indicated that soil TN, SOM, TC and S-ACP significantly influenced microbial diversity and C and P cycling-related genes. Intercropping with Perill reduced soil nutrients in the kiwifruit root zone, so there is a limitation to inter-cropping perilla in kiwifruit orchards, and this limitation should be considered in future field trials. Although only one growing season was intercropped in this study, we will extend the planting time in the future to try to make the inter-cropping assimilation more thorough. However, in the future research, I think we can combine the application of organic fertilizers in the process of intercropping perilla to make up for the soil nutrients consumed in the process, or adopt crop rotation to make up for the soil nutrients consumed in the perilla after harvesting, such as intercropping with leguminous plants, which can improve the nitrogen fertilizer of the soil, and explore a reasonable green intercropping system, in order to improve the utilization rate of the land and bring certain economic value to the farmers. The system will improve the utilization of land and bring some economic value to the farmers.

## Data availability statement

The datasets presented in this study can be found in online repositories. The names of the repository/repositories and accession number(s) can be found below: https://www.ncbi.nlm.nih.gov/sra/, PRJNA1077666.

## Author contributions

SZ: Writing – review & editing. NG: Writing – original draft. HW: Writing – review & editing. ZS: Writing – review & editing. YZ: Writing – review & editing. WZ: Writing – review & editing. YH: Writing – review & editing. SY: Writing – review & editing. HZ: Writing – review & editing. JQ: Writing – review & editing. WW: Writing – review & editing.
