# Supplementary material for: Macrogenomics reveal the effects of inter-cropping perilla on kiwifruit: impact on inter-root soil microbiota and gene expression of carbon, nitrogen, and phosphorus cycles in kiwifruit
Source: Front Microbiol. 2024 Jun 3;15:1349305. doi: 10.3389/fmicb.2024.1349305 (PMC11180754; doi:10.3389/fmicb.2024.1349305)

Supplementary Figures

Fig S1 NMDS display significant differences within and between Inter-root microbial communities. Circles of different colors indicate the distribution of microorganisms (fungi and bacteria) in different samples.

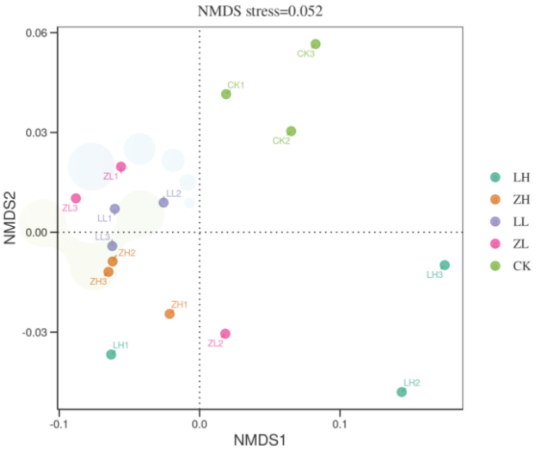

Fig S2 The principal coordinate analysis (PCoA) method based on Bray Curtis distance was used to determine the microbial functional profiles in the C **(A),** N **(B)**, and P **(C)** cycles of kiwifruit rhizosphere soil. CK: kiwifruit monoculture; LH: Qi Su 2+112500 plants/hm ^{2} ; LL: Qi Su 2+75000 plants/hm^{2} ZH: Gui Zi 2+112500 plants/hm^{2}; ZL: Gui Zi 2+112500 plants/hm^{2} .

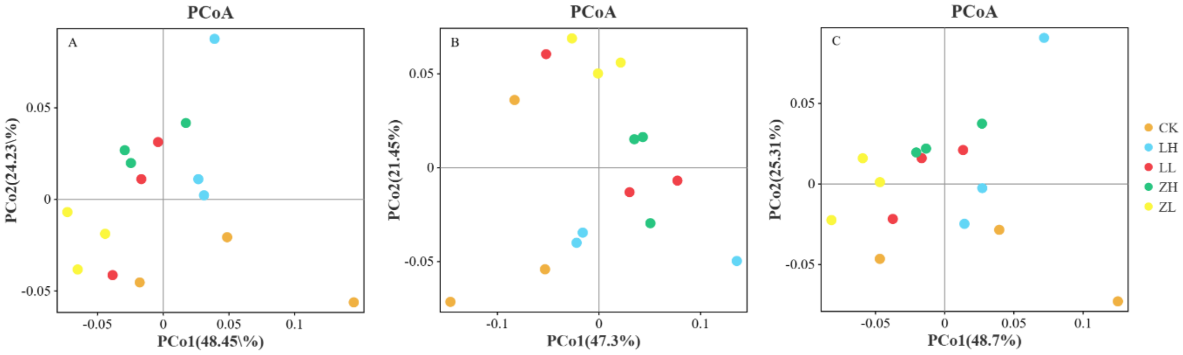

Supplement: Supplementary file 1 [file Data_Sheet_1.pdf]
